# The choroid as a vascular sensor in pulmonary hypertension: an oculomic mini review

**DOI:** 10.3389/fcvm.2026.1779239

**Published:** 2026-03-19

**Authors:** Diana A. Dmuchowska

**Affiliations:** Ophthalmology Department, Medical University of Bialystok, Bialystok, Poland

**Keywords:** choroid, choroidal vascularity index, choroidal thickness, oculomics, optical coherence tomography, pachychoroid-like, pulmonary hypertension

## Abstract

**Background:**

Pulmonary hypertension (PH) is increasingly recognized as a systemic vascular disorder with extrapulmonary end-organ involvement. Within oculomics, ocular imaging biomarkers are explored as systemic disease surrogates reflecting microvascular pathology. Owing to its high flow, fenestrated vasculature, and venous drainage pattern, the choroid is particularly susceptible to venous congestion and impaired ocular perfusion pressure. Optical coherence tomography (OCT) and OCT angiography (OCT-A) enable non-invasive quantitative assessment of choroidal structure, vascularity, and perfusion, thereby giving rise to candidate oculomic readouts*.*

**Methods:**

A narrative review of PubMed, Web of Science, and Scopus (through December 2025) identified clinical OCT/OCT-A studies and case reports on choroidal parameters in PH.

**Results:**

Due to concurrent, competing hemodynamic forces, two ends of a dynamic continuum of the choroidal end-organ phenotypes were identified. PH-targeted therapies and autonomic dysregulation may further modify this spectrum. A congestive pachychoroid-like phenotype, demonstrated in idiopathic pulmonary arterial hypertension, is characterized by pressure-dependent choroidal venous congestion, increased subfoveal choroidal thickness, and serous retinal detachment. An ischemic phenotype is characterized by choroidal thinning, perfusion defects, and structural remodeling. In chronic thromboembolic PH, OCT-A shows ischemic signatures with reduced vessel density and an enlarged foveal avascular zone. In precapillary PH, a prospective OCT study reveals reduced choroidal thickness and volume without consistent association with invasive cardiopulmonary hemodynamics.

**Conclusions:**

Choroidal oculomic candidate markers reflect microvascular remodeling in PH. Currently they lack sufficient validation as standalone surrogates of cardiopulmonary disease severity. Longitudinal multimodal imaging integrated with invasive hemodynamics is required to define their translational clinical utility.

## 1 Introduction

Pulmonary hypertension (PH) is a heterogeneous and progressive cardiopulmonary disorder defined by a resting mean pulmonary arterial pressure >20 mmHg and classified into five major groups: pulmonary arterial hypertension (PAH, Group 1), PH due to left heart disease (Group 2), PH due to lung diseases and/or hypoxia (Group 3), chronic thromboembolic PH (CTEPH, Group 4), and PH with unclear or multifactorial mechanisms (Group 5) (1, 2). The disease is characterized by pulmonary vascular remodeling, endothelial dysfunction, impaired nitric oxide signaling, and progressive right ventricular failure (1, 2). Among these phenotypes, PAH and CTEPH represent the most severe and prognostically unfavorable forms, associated with high morbidity and mortality despite contemporary targeted pharmacotherapy (2, 3).

Experimental and human histopathological studies indicate that PH induces profound microvascular remodeling extending beyond the pulmonary circulation and involving highly vascularized organs, including the uveal tract. Chronic elevation of venous pressure and impaired nitric oxide–dependent vasoregulation, as well as hypoperfusion, promote endothelial dysfunction, capillary rarefaction, and maladaptive vascular remodeling characterized by intimal thickening, smooth muscle cell hypertrophy, perivascular fibrosis, and luminal narrowing (4–6). In systemic venous hypertension, choroidal vessels demonstrate venous dilation, stromal edema, and increased interstitial fibrosis, accompanied by reduced capillary density and impaired autoregulatory capacity, resulting in chronic tissue hypoxia and ischemia-driven remodeling (7, 8). These microvascular changes may result in a spectrum of end-organ phenotypes, from venous congestion–driven pachychoroid-like remodeling to chronic hypoperfusion–related choroidal thinning.

Importantly, the choroid is particularly susceptible to venous outflow obstruction and elevated episcleral venous pressure owing to its high flow, fenestrated vasculature, and lack of lymphatic drainage. This facilitates sustained vascular engorgement, impaired fluid resorption, and low-grade inflammatory activation (7, 9). The eye, owing to its highly vascularized uveal tract and unique venous drainage through the ophthalmic and cavernous venous systems, is particularly vulnerable to systemic venous hypertension. Elevated episcleral venous pressure, impaired choroidal outflow, altered ocular perfusion pressure, and endothelial damage have been implicated in secondary glaucoma, uveal effusion, serous retinal detachment, and chronic choroidal ischemia (9–20).

Recent advances in enhanced depth imaging and swept-source optical coherence tomography (EDI-/SS-OCT) and OCT angiography (OCT-A) enable non-invasive quantitative assessment of choroidal structure and perfusion (21). Beyond thickness, the choroidal vascularity index (CVI) has emerged as a physiologically meaningful potential marker of choroidal vascular and stromal remodeling (22, 23). The concept of oculomics as a non-invasive readout of systemic microvascular dysfunction is supported by OCT-based choroidal parameters studies in other vascular systemic diseases, including systemic sclerosis and diabetes, where CVI and choroidal thickness/volume metrics capture vascular–stromal remodeling (24–26). These imaging-derived parameters are increasingly explored as candidate indicators of systemic vascular dysfunction. Importantly, Gu et al. (18) additionally documented a distinct congestive choroidal phenotype in idiopathic pulmonary arterial hypertension (IPAH), characterized by significantly increased subfoveal choroidal thickness, ocular venous congestion, and choroidal stasis driven by elevated episcleral and ocular venous pressure, thereby providing *in vivo* evidence for pressure-dependent choroidal remodeling in pulmonary vascular disease. However, the role of choroidal imaging as a potential biomarker of pulmonary vascular disease remains insufficiently defined. Notably, a recent prospective cross-sectional study in precapillary PH demonstrated reduced macular choroidal thickness and volume supporting a hypoperfusion-dominant choroidal phenotype without consistent correlations to invasive cardiopulmonary parameters, underscoring uncertainty regarding choroidal metrics utility and highlighting the need for longitudinal multimodal imaging approaches (12). Additional OCT/OCT-A evidence in CTEPH supports an ischemic retinochoroidal pattern (19), while contemporary systemic reviews emphasize ocular involvement as part of the broader extrapulmonary PAH phenotype and highlight oculomics as an emerging translational biomarker domain (20). This Mini Review synthesizes emerging evidence that PH is associated with a continuum of choroidal phenotypes and proposes a translational framework in which choroidal imaging might provide complementary, non-invasive vascular readouts in pulmonary vascular disease.

The spectrum of reported choroidal/ocular manifestations of PH is summarized in Table 1.

**Table 1:** Reported choroidal and ocular manifestations of pulmonary hypertension and associated imaging findings.

First author (year)	Study design	PH subtype	*N* (patients/eyes)	Imaging modality	Main choroidal/ocular findings	Key conclusions
Dmuchowska et al. (2015) (11)	Case report	PAH	1/2	SD-OCT; fundus photography; ocular US	Bilateral serous macular detachment with cystoid outer retinal edema; Elschnig-like choroidal lesions; conjunctival venous dilation; dynamic SRF fluctuations	Findings are consistent with a hemodynamic–choroidal axis in PAH; retinal fluid dynamics fluctuated with cardiopulmonary status and therapy adjustments
Zonenberg et al. (2025) (12)	Prospective cross-sectional	Precapillary PH	29/29 + 37/37 controls	SD-OCT with EDI; CVI binarization	↓ CT and choroidal volume across ETDRS fields; CVI unchanged	Choroidal thinning may reflect chronic downstream microvascular remodeling/hypoperfusion; correlations with invasive hemodynamics largely non significant
Lei et al. (2019) (10)	Case report	Secondary PH (VSD-related)	1/2	OCT; UBM; US (B-scan); fundus photography	Dilated episcleral veins; elevated IOP with secondary angle-closure mechanism; transient myopic shift; 360° ciliochoroidal detachment; SRF on macular OCT	Secondary PH may cause venous outflow impairment with exudative ciliochoroidal detachment and secondary IOP elevation; ocular findings improved after systemic treatment
Saran et al. (2001) (13)	Retrospective case series	Familial IPAH	3/6	FA; ICG videoangiography (subset)	Delayed choroidal filling; choroidal perfusion defects; deep choroidal vessel dilation; choroidal detachment (subset)	Venous hypertension may impair choroidal perfusion causing delayed filling and detachments
Krohn & Bjune (2003) (14)	Case report	IPAH	1/2	Fundus exam; US (A/B-scan); MRI	Bilateral annular ciliochoroidal detachment; uveal effusion; intermittent secondary angle-closure glaucoma	Venous congestion in IPAH may induce uveal effusion with secondary angle-closure
Martiano et al. (2017) (15)	Case report	IPAH	1/1	FA; ICG; SD-OCT	Unilateral serous macular detachment; delayed choroidal filling; multiple perfusion defects; thick choroid (CT ≈600 µm); spontaneous resolution	PAH may present with transient mixed congestive–ischemic retinochoroidal phenotype
Matieli et al. (2016) (16)	Case–control study	PAH on sildenafil	20/40 vs. 20/40 controls	Clinical exam; TD/SD-OCT; fundus photos; color Doppler US (± ERG)	Severe keratitis; reduced BUT; ↓ resistance index in ophthalmic & posterior ciliary arteries	Chronic sildenafil associated with ocular surface morbidity and measurable retrobulbar hemodynamic effects
Wirostko et al. (2012) (17)	Phase III RCT + extension	PAH	277/277	Slit-lamp; funduscopy; IOP; visual function tests	Dilated episcleral vessels and conjunctival injection common; objective ocular parameters stable	Chronic sildenafil shows favorable ocular safety profile
Gu et al. (2021) (18)	Observational cohort	IPAH	22/44 + 22/44 controls	OCT-A	↓ macular vessel density (superficial/deep); ↓ RPC density; RNFL/GCC thinning with ↑ FLV/GLV; ↑ subfoveal CT; CC flow area not significantly different; fundus changes in subset	OCT-A detects retinal and neurovascular impairment, including preclinical changes
Krajewski et al. (2022) (19)	Observational cohort	CTEPH	36/72 + 65/130 controls	OCT; OCT-A	↓ deep vascular complex density; ↑ FAZ; ↓ subfoveal CT; ↓ RPC density with comorbidity	Retinochoroidal microvascular impairment is measurable in CTEPH
Lewczuk et al. (2019) (9)	Narrative review	Mixed PH	–	Review	Venous congestion, uveal effusion, serous RD, macular edema, CRVO, angle-closure glaucoma, transient myopia	Foundational synthesis of ocular PH manifestations
Singh et al. (2024) (20)	Expert review	PAH (extrapulmonary)	–	Review	Notes emerging oculomics concepts; retinal vascular pathology and venous congestion	Highlights need for standardized longitudinal oculomic studies

Across studies, reported choroidal directionality (thickening vs. thinning) likely reflects phenotype spectrum, PH etiology, comorbidity burden, imaging modality, and vasodilator modification. BUT, tear break-up time; CC, choriocapillaris; CRVO, central retinal vein occlusion; CSC, central serous chorioretinopathy; CT, choroidal thickness; CTEPH, chronic thromboembolic pulmonary hypertension; CVI, choroidal vascularity index; EDI, enhanced depth imaging; ERG, electroretinography; ETDRS, Early Treatment Diabetic Retinopathy Study; FA, fluorescein angiography; FAZ, foveal avascular zone; FLV, focal loss volume; GCC, ganglion cell complex; GLV, global loss volume; ICG, indocyanine green angiography; IOP, intraocular pressure; IPAH, idiopathic pulmonary arterial hypertension; MRI, magnetic resonance imaging; OCT, optical coherence tomography; OCT-A, optical coherence tomography angiography; PAH, pulmonary arterial hypertension; PDE-5i, phosphodiesterase-5 inhibitor; PH, pulmonary hypertension; RCT, randomized controlled trial; RD, retinal detachment; RNFL, retinal nerve fiber layer; RPC, radial peripapillary capillary; SD-OCT, spectral-domain optical coherence tomography; SRF, subretinal fluid; SS-OCT, swept-source optical coherence tomography; TD-OCT, time-domain optical coherence tomography; UBM, ultrasound biomicroscopy; US, ultrasonography; VSD, ventricular septal defect.

## 2 Search methodology

A narrative literature search was conducted in PubMed, Web of Science, and Scopus for articles published up to December 2025. Three complementary PubMed queries (through December 2025) were used:

(1) to inform cardiological context, pulmonary hypertension (PH) background literature was identified using a broad PH-focused PubMed query. Given the very large yield (>∼17,000 records), this component was used for narrative contextualization rather than quantitative evidence synthesis. In addition, the ophthalmic imaging framework was informed by reviews on oculomics, choroid and the CVI, which were used to contextualize the present narrative review.
(2) a PH ocular/imaging query, which retrieved 191 records: PubMed query: (“Hypertension, Pulmonary” OR “pulmonary hypertension” OR “pulmonary arterial hypertension” OR “chronic thromboembolic pulmonary hypertension” OR PAH OR CTEPH OR “precapillary pulmonary hypertension”) AND (ocular OR eye OR retina* OR retinal OR choroid* OR choroidal OR “uveal effusion” OR “choroidal detachment” OR ciliochoroidal OR “serous retinal detachment” OR “subretinal fluid” OR “episcleral venous” OR “venous congestion” OR “angle-closure” OR “intraocular pressure”) AND (“Tomography, Optical Coherence” OR OCT OR “optical coherence tomography” OR OCTA OR “OCT angiography” OR “optical coherence tomography angiography” OR EDI OR “enhanced depth imaging” OR “swept-source” OR “swept source” OR “SS-OCT” OR “SS-OCTA” OR “fluorescein angiography” OR FA OR “indocyanine green” OR ICG OR ultrasonography OR ultrasound OR UBM)
(3) a drug query (eg PDE-5 inhibitors and riociguat) which retrieved 180 records. PubMed query: (sildenafil OR “Sildenafil Citrate” OR tadalafil OR “Tadalafil” OR vardenafil OR avanafil OR riociguat OR “Riociguat” OR “phosphodiesterase 5” OR phosphodiesterase-5 OR PDE5 OR “PDE-5” OR PDE-5i OR “soluble guanylate cyclase” OR sGC OR bosentan OR “Bosentan” OR ambrisentan OR “Ambrisentan” OR macitentan OR “Macitentan” OR “endothelin receptor antagonist” OR epoprostenol OR “Epoprostenol” OR treprostinil OR “Treprostinil” OR iloprost OR “Iloprost” OR selexipag OR “Selexipag” OR “prostacyclin”) AND (choroid* OR choroidal OR retina* OR retinal OR episcleral OR conjunctiva* OR fundus) AND (“optical coherence tomography” OR OCT OR OCTA OR “OCT angiography” OR “optical coherence tomography angiography” OR “enhanced depth imaging” OR EDI OR “swept-source” OR “swept source” OR “SS-OCT” OR “blood flow” OR perfusion OR “ocular perfusion” OR “retinal blood flow” OR “choroidal blood flow” OR “intraocular pressure” OR IOP OR Doppler OR “color Doppler” OR electroretinogram OR ERG OR “visual field”) NOT (“erectile dysfunction” OR impotence)

Equivalent keyword strategies were applied in Web of Science and Scopus. Records were screened at title/abstract level, followed by full-text eligibility assessment. After deduplication across queries and exclusion of non-relevant articles, 43 references were retained for citation, including 12 studies summarized in Table 1.

Original studies, case reports and case series were included. Animal studies were included but were cited selectively to support pathophysiological interpretation. Conference abstracts without full-text availability and pediatric populations were excluded.

## 3 Choroidal phenotypes of PH

In PH, choroidal structure is likely shaped by concurrent, competing hemodynamic forces rather than a single-direction pathway. Reduced anterograde perfusion pressure and endothelin-1-driven precapillary vasoconstriction may promote choroidal capillary rarefaction and remodeling, whereas retrograde transmission of systemic venous congestion can elevate ocular venous pressure and favor venous stasis and stromal expansion. PH-targeted therapies and autonomic dysregulation are plausible biological modifiers. Therefore, the choroidal phenotypes of PH should be interpreted as dynamic ends of a continuum, and mixed phenotypes may occur depending on the balance between inflow limitation and outflow obstruction at a given timepoint.

### 3.1 Congestive pachychoroid-like phenotype

This phenotype is mechanistically linked to chronic venous congestion, elevated right atrial pressure, and impaired ocular venous outflow through the vortex veins, leading to increased hydrostatic pressure, vascular dilation, and stromal edema (7–10, 14).

Clinical and imaging evidence indicates that PH can induce a congestive pachychoroid-like phenotype characterized by choroidal thickening, stromal expansion, and venous congestion (7, 9–11, 14, 15, 18). The term “pachychoroid” in this context refers to a morphological phenotype of the choroid, rather than implying the full pachychoroid disease spectrum. Observed pachychoroid-like features in PH probably primarily reflect hemodynamic and microvascular remodeling rather than a distinct ophthalmologic pachychoroid disease entity. This pattern has been demonstrated *in vivo* using OCT and OCT-A in IPAH by Gu et al. (18), who reported significantly increased subfoveal choroidal thickness (SFCT), choroidal venous dilation, and choroidal stasis compared with healthy controls. These changes might be occasionally accompanied by serous retinal detachments, thereby providing quantitative evidence for pressure-dependent pachychoroid remodeling in pulmonary vascular disease. In addition, clinically, this phenotype may manifest as central serous chorioretinopathy–like or uveal effusion–related changes, as well as angle closure glaucoma, in selected cases in idiopathic and secondary PH. Nevertheless, current evidence does not support a generalized increased risk of pachychoroid spectrum diseases in PH populations (9–11, 14, 15). A paradigmatic example was reported in a patient with PAH treated with chronic phosphodiesterase-5 inhibition (PDE-5) who developed bilateral serous macular detachment, Elschnig-like lesions, and cystoid retinal edema, with dynamic subretinal fluid fluctuations paralleling cardiopulmonary status, providing clinical support for a hemodynamic–choroidal axis (11). Pharmacological modulation of the nitric oxide–cyclic guanosine monophosphate (NO–cGMP) pathway, particularly through chronic PDE-5 inhibition, may act as a biological modifier by augmenting precapillary inflow and choroidal blood volume (17, 27–30). Therefore, reports defining a congestive pachychoroid-like phenotype should explicitly account for phosphodiesterase-5 inhibitors (PDE-5i) exposure and, where possible, separate therapy-naïve from therapy-modified states.

However, OCT and OCT-A evidence from IPAH supports pressure-dependent choroidal venous stasis beyond isolated drug effects (18), and choroidal thinning has also been reported in precapillary PH despite PDE-5i exposure in a substantial proportion of patients (12). In the absence of therapy-naïve longitudinal data, definitive causal attribution remains limited. Thus, PDE-5 inhibition should be regarded as a modifying, rather than primary, explanatory factor within a broader hemodynamic framework.

### 3.2 Ischemic choroidopathy phenotype

An ischemic choroidopathy phenotype has also been described in PH, characterized by delayed choroidal filling, perfusion defects, and chorioretinal ischemic changes (8, 9, 13, 15). In addition to anterograde hypoperfusion, endothelin-1–mediated vasoconstriction and impaired autoregulation further predispose the choroid to ischemic injury in PH (31–33).

In CTEPH, OCT-A studies demonstrate reduced macular and peripapillary vessel density metrics, enlargement of the foveal avascular zone (FAZ), and reduced mean subfoveal choroidal thickness, supporting a retinal–choroidal ischemic signature in this phenotype (19).

Particularly, an OCT-based study suggests that precapillary PH may be associated with choroidal thinning rather than thickening, potentially reflecting chronic hypoperfusion and long-term microvascular remodeling. In a prospective cross-sectional cohort, both choroidal thickness and volume were significantly reduced in precapillary PH compared with controls, whereas the CVI, a quantitative marker of choroidal angioarchitecture, remained unchanged, indicating structural thinning with proportional luminal involvement (12, 22).

In parallel, autonomic dysregulation and sympathetic activation can influence systemic hemodynamics (heart rate, cardiac output) and peripheral vascular tone, while also modulating choroidal vasomotor control. Together, these effects may reduce ocular perfusion pressure and bias the net phenotype toward inflow limitation/hypoperfusion or mixed presentations (5, 8).

## 4 Hemodynamic–choroidal axis

Evidence linking invasive cardiopulmonary hemodynamics with OCT-derived choroidal parameters remains limited and inconsistent. In the largest prospective cross-sectional OCT study to date, precapillary PH was associated with significantly reduced choroidal thickness and volume, whereas the CVI did not differ between patients and controls and no consistent associations were identified between most cardiological variables and choroidal metrics (12). In contrast, OCT and OCT-A evidence from IPAH demonstrates that elevated pulmonary arterial and systemic venous pressures propagate to increased episcleral and ocular venous pressure, resulting in choroidal venous stasis and significant subfoveal choroidal thickening, thereby suggesting a pressure-dependent hemodynamic–choroidal axis (18). Complementary OCT-A evidence in CTEPH indicates that microvascular parameters, namely FAZ and vessel density in superficial/deep macular plexuses and radial peripapillary capillary plexus, vary with comorbidity burden, suggesting that systemic vascular disease may modify the ocular hemodynamic signature beyond PH-specific mechanisms (19).

**Figure 1:**
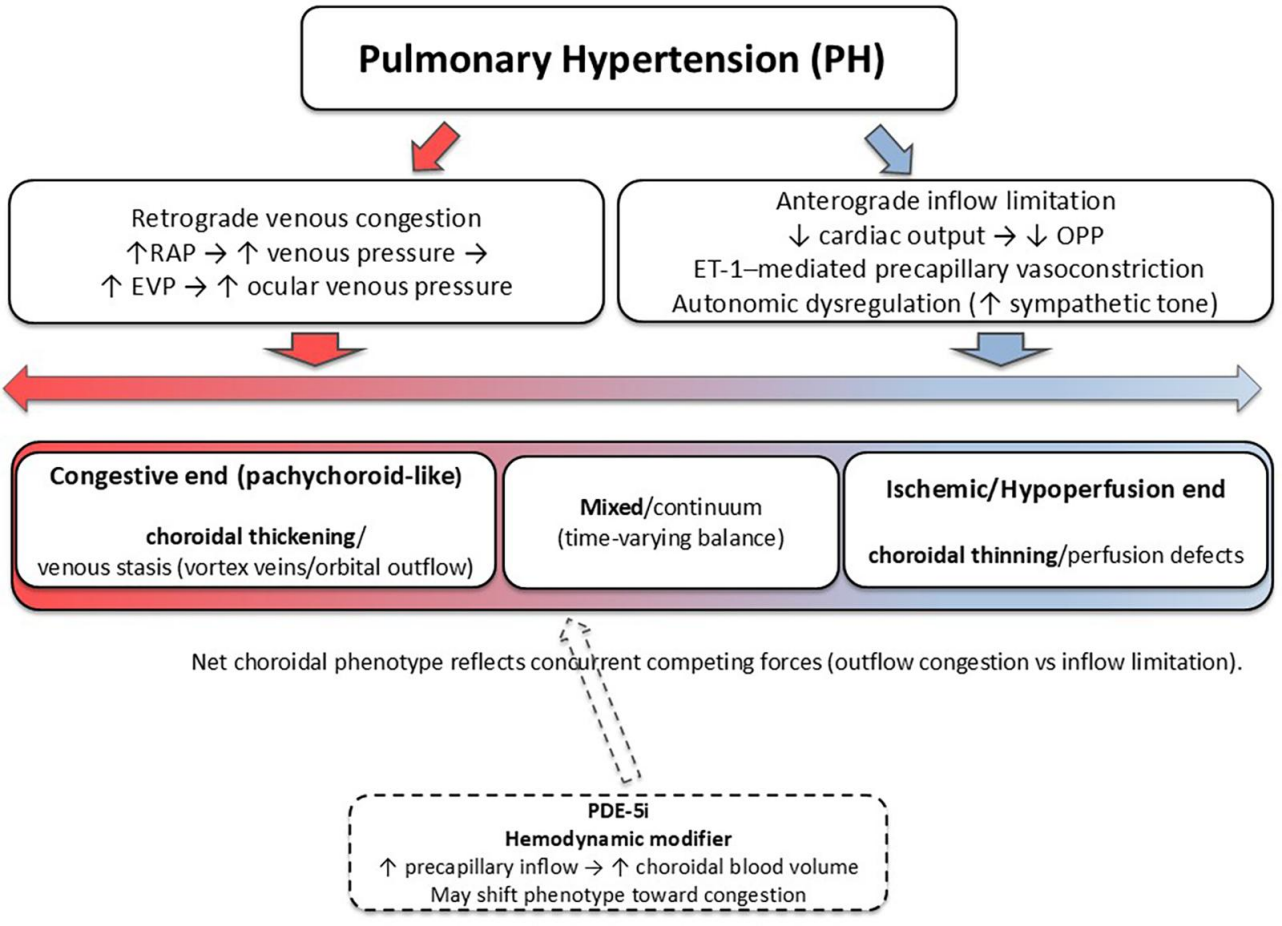
Hemodynamic–choroidal axis in pulmonary hypertension: competing forces, phenotypic continuum, and therapy modifiers. Proposed conceptual model linking PH to choroidal remodeling through simultaneous, opposing hemodynamic mechanisms. The net choroidal phenotype emerges from the balance between (1) venous outflow impairment with elevated ocular venous pressure/EVP (congestion, favoring pachychoroid-like thickening) and (2) reduced effective OPP due to reduced cardiac output and ET-1–mediated precapillary vasoconstriction (ischemia/hypoperfusion, favoring choroidal thinning). Mixed phenotypes may occur when both forces are present. PH-targeted therapies, particularly PDE-5i, are shown as biological modifiers that can shift the apparent phenotype by augmenting precapillary inflow in the setting of venous outflow limitation. Autonomic dysregulation (sympathetic predominance) may further modulate this axis through effects on cardiac output, systemic vascular tone (and thus OPP), and choroidal vasomotor tone, thereby biasing the net phenotype toward hypoperfusion or mixed presentations. ET-1, endothelin-1; EVP, episcleral venous pressure; OPP, ocular perfusion pressure; PDE-5i, phosphodiesterase-5 inhibitor; RAP, right atrial pressure.

From a pathophysiological perspective, elevated right atrial pressure and retrograde systemic venous congestion propagate to the ocular venous circulation through the vortex and ophthalmic veins, increasing choroidal hydrostatic pressure and impairing ocular perfusion pressure (7–10, 14). This hemodynamic environment has been quantitatively linked to choroidal venous congestion and pachychoroid-like remodeling *in vivo* in PAH (18). On the other hand, anterograde inflow limitation due to reduced effective ocular perfusion pressure and endothelin-1–mediated precapillary vasoconstriction opposes the venous congestion. This ischemic signature is further supported by historical angiography (13, 15). Consequently, the net choroidal phenotype reflects their time-varying dynamic balance on the continuum of choroidal end-organ responses, from congestive pachychoroid-like remodeling to ischemic choroidopathy (7–9, 23). These phenotypes likely represent a spectrum rather than mutually exclusive states with pharmacologic modulation (e.g., PDE-5 inhibition) and sympathetic activation acting as modifying factors. The proposed hemodynamic–choroidal axis in PH is summarized as a conceptual framework in Figure 1.

Similar choroidal OCT/OCT-A alterations have been demonstrated in heart failure, consistent with the choroid acting as a vascular sensor of central hemodynamics (34). Collectively, current data indicate a potential role for the choroid as a vascular sensor in pulmonary vascular disease, but its standalone biomarker utility remains limited.

## 5 Therapy-related choroidal remodeling

Targeted PH therapies exert clinically relevant effects on choroidal structure and perfusion, with the most consistent ocular signal observed for PDE-5i. In a prospective OCT-based cohort of patients with PAH, chronic sildenafil therapy was associated with increased choroidal thickness and altered ocular perfusion, providing the only available *in vivo* human evidence for therapy-related choroidal remodeling in PH (16).

Case-based and cohort observations further describe pachychoroid- and central serous chorioretinopathy–like phenotypes temporally associated with chronic PDE-5i exposure in PAH, particularly in patients receiving higher cumulative doses (10, 11, 16). OCT/OCT-A studies in healthy volunteers demonstrate acute sildenafil-induced increases in choroidal thickness and choroidal blood volume, supporting a vasoactive basis for these effects (27, 30).

For tadalafil, transient increases in choroidal thickness and retinal microvascular density have been reported shortly after treatment initiation, with partial attenuation over time, suggesting an initial hemodynamic effect that may normalize during chronic exposure (35).

For endothelin receptor antagonists, prostacyclin-pathway therapies, and soluble guanylate cyclase stimulators, direct PH–specific OCT/OCT-A data on choroidal structure remain limited. Nevertheless, mechanistic plausibility is strong, as endothelin-1 critically regulates retinal and choroidal blood flow (32, 33, 36). Prostacyclin analogues modulate choroidal vascular tone via prostanoid receptor–mediated vasodilation, and soluble guanylate cyclase stimulation augments cGMP signaling (37). Among guanylate cyclase stimulators, riociguat has demonstrated clinical efficacy in PAH and CTEPH (3, 37), although systematic OCT/OCT-A choroidal endpoints have not yet been evaluated in PH cohorts.

Importantly, interpretation of choroidal thickening vs. thinning across OCT/OCT-A studies should account for PH etiology and exposure to vasodilator therapy, particularly PDE-5i, which can introduce etiology-specific confounding by indication. A key limitation of the current literature is that several reports include PDE-5i-exposed rather than treatment-naïve patients, which may lead to confounding.

## 6 Candidate oculomic markers and their potential clinical translation

Modern imaging enables non-invasive quantitative assessment of choroidal structure and perfusion. Within the emerging concept of oculomics, candidate ocular imaging metrics are increasingly explored as systemic disease surrogates reflecting microvascular pathology beyond the eye (38). Beyond conventional thickness measurements, the CVI provides a more stable indicator of choroidal angioarchitecture and stromal–luminal remodeling, being less affected by age, axial length, and diurnal variation (22, 39). OCT-A further allows assessment of both retinal and choriocapillaris microvascular integrity through macular and peripapillary vessel density and foveal metrics (7, 40). The biological plausibility of choroidal imaging-derived metrics in PH is supported by OCT and OCT-A evidence of pressure-dependent choroidal venous congestion and subfoveal choroidal thickening in IPAH (18). Importantly, OCT-A data in CTEPH demonstrate reduced macular and peripapillary vessel density, FAZ enlargement, and reduced subfoveal choroidal thickness, with modification by comorbidity burden, suggesting potential clinically relevant microvascular phenotypes (19).

Nevertheless, current clinical validity remains limited. Among available OCT-based studies only Zonenberg et al. systematically correlated choroidal parameters with invasive cardiopulmonary hemodynamics (12). This highlights the need for longitudinal, etiology-stratified, and treatment-aware oculomic studies integrating ocular imaging with invasive and clinical endpoints.

## 7 Knowledge gaps and future directions

Current evidence is largely derived from case reports and small observational studies, limiting reliable prevalence estimates and prognostic analyses. Major gaps include the lack of standardized imaging protocols and the limited integration of complementary choroidal phenotyping tools with invasive cardiopulmonary hemodynamic data to validate choroidal metrics across distinct PH types. Future prospective translational studies should adopt automated standardized multimodal imaging protocols, including EDI- or swept-source OCT–derived choroidal thickness and CVI, combined with OCT-A macular and peripapillary vessel density metrics. Most of the available studies utilized SD-OCT/SD-OCT-A, which provides less reliable deep choroidal visualization than swept-source OCT. Although some studies used EDI to improve choroidal delineation, it does not fully overcome depth-related signal attenuation. Key confounders such as diurnal variation, axial length, hydration status, autonomic tone, and ocular perfusion pressure should be systematically controlled. They should additionally incorporate detailed stratification by pulmonary hypertension–targeted therapies, particularly PDE-5i, including treatment duration, dosing, and timing relative to ocular imaging. Longitudinal designs integrating ocular imaging with right-heart catheterization, echocardiography, and validated cardiopulmonary endpoints [N-terminal pro–B-type natriuretic peptide [NT-proBNP], 6-minute walk distance, and World Health Organization [WHO] functional class] are required to determine whether choroidal phenotypes represent stable disease traits, reflect therapy-responsive microvascular remodeling, or serve as dynamic indicators of cardiopulmonary decompensation. Therapeutic interventions should be leveraged primarily as biological perturbation models to test choroidal metrics responsiveness and enable risk stratification and prognostic modeling, rather than as primary clinical outcomes (1, 3).

## 8 Discussion

This Mini Review frames PH as a systemic vascular disorder in which choroidal remodeling spans a dynamic continuum, from congestive pachychoroid-like phenotype to ischemic choroidal phenotype. The juxtaposition of case-based literature suggesting pachychoroid-like congestive remodeling (10, 11, 14, 15) with prospective cross-sectional data demonstrating reduced choroidal thickness and volume without consistent hemodynamic correlations (12) underscores a fundamental controversy regarding the direction of remodeling and the need for definitive validation before any biomarker claims. Importantly, morphological pachychoroid-like remodeling should be distinguished from pachychoroid spectrum diseases, which require additional evidence of retinal pigment epithelium dysfunction, subretinal fluid, or characteristic angiographic features. Beyond the choroid, OCT-A evidence demonstrates retinochoroidal microvascular alterations in PAH and CTEPH that reflect systemic endothelial dysfunction and vascular remodeling, further supporting the concept of PH as a systemic vasculopathy rather than a lung-limited disease (18, 19). Importantly, *in vivo* OCT and OCT-A evidence from IPAH demonstrates that elevated pulmonary and systemic venous pressures propagate to increased episcleral and ocular venous load, resulting in choroidal venous stasis and pressure-dependent subfoveal choroidal thickening, suggesting a possible link to a congestive pachychoroid-like phenotype presentation, while acknowledging that therapy exposure and mixed phenotypes may modify the observed morphology (18). The coexistence of neural layer thinning and retinal capillary rarefaction suggests concomitant neurodegenerative processes secondary to chronic hypoxia and microvascular insufficiency (18), indicating that ocular involvement in PH extends to both vascular and neuroretinal compartments. The largest OCT/OCT-A cohort in CTEPH characterized retinochoroidal microvascular impairment primarily through between-group comparisons and comorbidity stratification, without evaluating correlations between ocular metrics (vessel density, FAZ, and subfoveal choroidal thickness) and invasive cardiopulmonary hemodynamic parameters, leaving the choroidal–hemodynamic coupling unresolved (19).

In this context, Singh et al. (20) provide a contemporary systemic framework linking extrapulmonary manifestations of PAH to inflammatory and metabolic dysregulation, strengthening the biological plausibility of widespread ocular microvascular and neuroretinal involvement. Collectively, these data position OCT and OCT-A as promising research tools for phenotyping and longitudinal characterization of ocular microvascular remodeling in PAH and CTEPH. Any potential role in early detection, monitoring, or risk stratification remains hypothesis-generating. Based on current evidence their role should be regarded as complementary rather than definitive.

At present, we cannot separate natural disease trajectory from pharmacologically modified hemodynamics. Pathophysiologically, ocular phenotypes likely reflect the balance between venous backpressure and impaired ocular perfusion pressure in a setting of systemic endothelial dysfunction and pharmacologic modulation (7, 8, 10). Chronic exposure to PAH therapies, particularly PDE-5i, may further modify choroidal vascular tone and permeability, potentially amplifying congestive phenotypes in susceptible individuals (27, 30, 41). These therapy-related effects highlight the importance of stratifying future studies by PH etiology and pharmacotherapy exposure.

From a translational perspective, choroidal and retinal parameters offer an attractive, non-invasive approach to systemic vascular phenotyping. However, current evidence does not support the use of OCT- or OCT-A–derived parameters as reliable standalone markers of pulmonary vascular disease severity. Rather, ocular imaging may provide complementary insights into microvascular and stromal remodeling in PH.

Future prospective studies should prioritize standardized multimodal imaging protocols, longitudinal designs, and integration with invasive hemodynamic measurements and validated cardiopulmonary outcomes (1). Such approaches will be essential to determine whether ocular phenotypes represent stable disease traits, treatment-related effects, or dynamic indicators of cardiopulmonary decompensation, and to define their translational clinical utility.

## 9 Conclusions

PH is associated with a spectrum of choroidal changes ranging from congestive pachychoroid-like to ischemic choroidal end-organ phenotypes. They are driven by concurrent, competing forces of retrograde venous congestion, anterograde hypoperfusion, endothelial dysfunction, and modifying effects of PDEi. Emerging multimodal OCT-based imaging enables objective, quantitative assessment of these phenotypes and supports the concept of the choroid as a potential vascular sensor of pulmonary vascular disease.

*In vivo* OCT and OCT-A evidence demonstrates pressure-dependent choroidal venous congestion and subfoveal choroidal thickening in IPAH (18). To date, the largest available OCT/OCT-A cohort in PH, comprising CTEPH, demonstrates significant retinal and choroidal microvascular impairment, including reduced macular and peripapillary vessel density, enlargement of the FAZ, and reduced choroidal thickness (19). Importantly, this study did not evaluate associations with invasive hemodynamic parameters, precluding direct inference regarding choroidal–hemodynamic association. In contrast, the only prospective OCT study that systematically integrated choroidal imaging with right-heart catheterization parameters demonstrated reduced choroidal thickness and volume in precapillary PH without consistent correlations to invasive cardiopulmonary metrics (12), indicating that choroidal metrics should currently be regarded as potential and complementary rather than reliable standalone biomarkers of pulmonary vascular disease severity.

Choroidal imaging offers a unique, non-invasive window into microvascular remodeling that is otherwise inaccessible to routine cardiopulmonary assessment. Longitudinal multimodal imaging studies integrating ocular metrics with invasive cardiopulmonary hemodynamics and clinical outcomes are therefore required to define the translational utility of choroidal oculomic parameters in PH.
